# Systems analysis reveals alternate metabolic states adopted by *Mycobacterium tuberculosis* across species

**DOI:** 10.1128/msphere.00141-26

**Published:** 2026-06-29

**Authors:** Jonathan Padilla-Gomez, Rachel McGinn, Victoria De Andrade, Yisha Liang, Kendra Libby, Heyuan Michael Ni, Alison E. Ringel, Charles L. Evavold, Bryan D. Bryson

**Affiliations:** 1 Department of Biological Engineering, Massachusetts Institute of Technology 196195, Cambridge, Massachusetts, USA; 2 Ragon Institute of Mass General, MIT and Harvard 200750 , Cambridge, Massachusetts, USA; 3 Department of Biology, Massachusetts Institute of Technology 311383, Cambridge, Massachusetts, USA; University of Minnesota Twin Cities, Minneapolis, Minnesota, USA

**Keywords:** *Mycobacterium tuberculosis*, innate immunity, macrophages

## Abstract

*Mycobacterium tuberculosis* (Mtb) survives within macrophages by adapting its metabolism to host-imposed constraints, yet how host species shape bacterial metabolic state remains poorly defined. Here, we directly compared the intracellular transcriptional and metabolic states of Mtb during infection of human and mouse macrophages. Cross-species transcriptomics revealed distinct host-imposed microenvironments that drive Mtb into separable metabolic programs, with mouse macrophages inducing stress-associated pathways and human macrophages promoting fatty acid import and catabolism. Strikingly, these differences manifested in a species-specific phenotype: Mtb formed intracellular lipid inclusions (ILIs) in murine macrophages but not in human macrophages, despite equivalent lipid availability. This divergence was independent of macrophage ontogeny, culture conditions, nitric oxide, or itaconate. Instead, bacterial lipid storage in ILIs was inversely correlated with host triacylglycerol synthesis. Pharmacologic inhibition of diacylglycerol acyltransferase 1 in human macrophages partially restored Mtb ILI formation, identifying host lipid sequestration as a metabolic gate that restricts bacterial lipid storage. Together, these findings establish host lipid metabolism as a key determinant of intracellular Mtb metabolic state and reveal a species-specific barrier that limits bacterial access to host lipid stores in human macrophages.

**IMPORTANCE:**

Tuberculosis remains a leading cause of infectious death worldwide, yet much of what we know about how *Mycobacterium tuberculosis* survives inside immune cells comes from studies in animal models. This work shows that human and mouse macrophages impose fundamentally different metabolic constraints on the bacterium, leading to distinct survival strategies. In mouse cells, the pathogen adopts a stress-associated state and stores lipids, whereas in human cells, it does not. We identify host lipid metabolism as a key factor limiting bacterial access to these resources in human macrophages. These findings highlight an important species-specific difference that may influence how well animal models predict human infection and suggest that targeting host lipid pathways could offer new strategies to control tuberculosis.

## INTRODUCTION

*Mycobacterium tuberculosis* (Mtb), the causative agent of tuberculosis, remains one of the world’s leading infectious killers. Mtb’s ability to persist within macrophages is central to its success as a pathogen, yet the physiological states it adopts in different host environments remain incompletely defined. Mouse models have been indispensable for uncovering fundamental principles of Mtb–host interactions because of their genetic tractability, experimental reproducibility, and suitability for *in vivo* studies. However, it is well appreciated that key immunologic and metabolic features differ between murine and human macrophages, raising questions about how these differences shape Mtb physiology.

Comparative studies of species differences have largely focused on divergent host responses to infection rather than on the consequences for the bacterium itself. Two canonical examples, *nos2*, encoding inducible nitric oxide synthase, and *irg1*, which produces the antimicrobial metabolite itaconate, illustrate how the macrophage’s antimicrobial landscape differs across species (1–4). Both are robustly induced in mouse macrophages but are more limited in human cells, resulting in distinct patterns of immune pressure. Yet how Mtb adapts metabolically and transcriptionally to these species-specific environments remains poorly understood.

Beyond direct antimicrobial stress, host cells impose nutritional constraints that strongly influence Mtb survival. Nutrient limitation represents a major axis of bacterial vulnerability, and Mtb’s capacity to remodel its metabolism under such conditions is a major determinant of persistence. Mtb’s strategies for nutrient acquisition, including fatty acid and cholesterol uptake, have therefore emerged as central to its pathogenesis and as potential therapeutic targets. For example, loss of the Mce4 transporter impairs cholesterol utilization and restricts growth in activated mouse macrophages (5), underscoring how metabolic interplay between host and pathogen dictates infection outcomes.

Here, we apply a quantitative, cross-species framework to directly compare Mtb physiology in human and mouse macrophages. This analysis reveals extensive differential gene expression, highlighting distinct bacterial metabolic programs across hosts. Functional and metabolic validation identified a striking difference in the formation of intracellular lipid inclusions (ILIs), neutral lipid stores associated with metabolic quiescence and stress tolerance (6, 7) in Mtb. We further show that modulation of triacylglycerol (TAG) biosynthesis in human macrophages alters Mtb ILI formation, defining a metabolic coupling that links host lipid flux to bacterial lipid storage.

These findings demonstrate that host species-specific environments drive distinct bacterial physiological states and uncover a previously unrecognized connection between host lipid droplets (LDs) and Mtb lipid inclusions. More broadly, our cross-species approach provides a framework for dissecting how host context shapes pathogen state, offering a path toward more predictive models of Mtb pathogenesis.

## RESULTS

### Host species shape *M. tuberculosis* transcriptional programs in macrophages

Macrophages constitute the principal cellular niche for Mtb and simultaneously mediate antimicrobial control. Foundational insights into host–Mtb interactions have come from *in vitro* infection models in which macrophages are sampled for immunologic or microbiologic analyses. Mouse macrophages are widely used, yet several studies have underscored the limits of extrapolating antimicrobial mechanisms from mice to humans. For example, the contribution of reactive nitrogen species remains debated (8, 9), and recent work points to species differences in the antimicrobial metabolite itaconate arising from a human-specific variant in aconitate decarboxylase 1 (10). These observations highlight a gap in our understanding of how macrophage models behave across species in the context of Mtb infection.

Most cross-species comparisons have profiled the host response to defined stimuli (e.g., Toll-like receptor ligands) or infection, revealing differences in the magnitude and quality of host pathways (8, 11, 12). Here, we instead asked how Mtb changes state in distinct host macrophage environments. Measuring the Mtb transcriptome has proven informative for comparing macrophage activation states and Mtb genetic perturbations (13, 14); we applied the same strategy across species.

**Fig 1:**
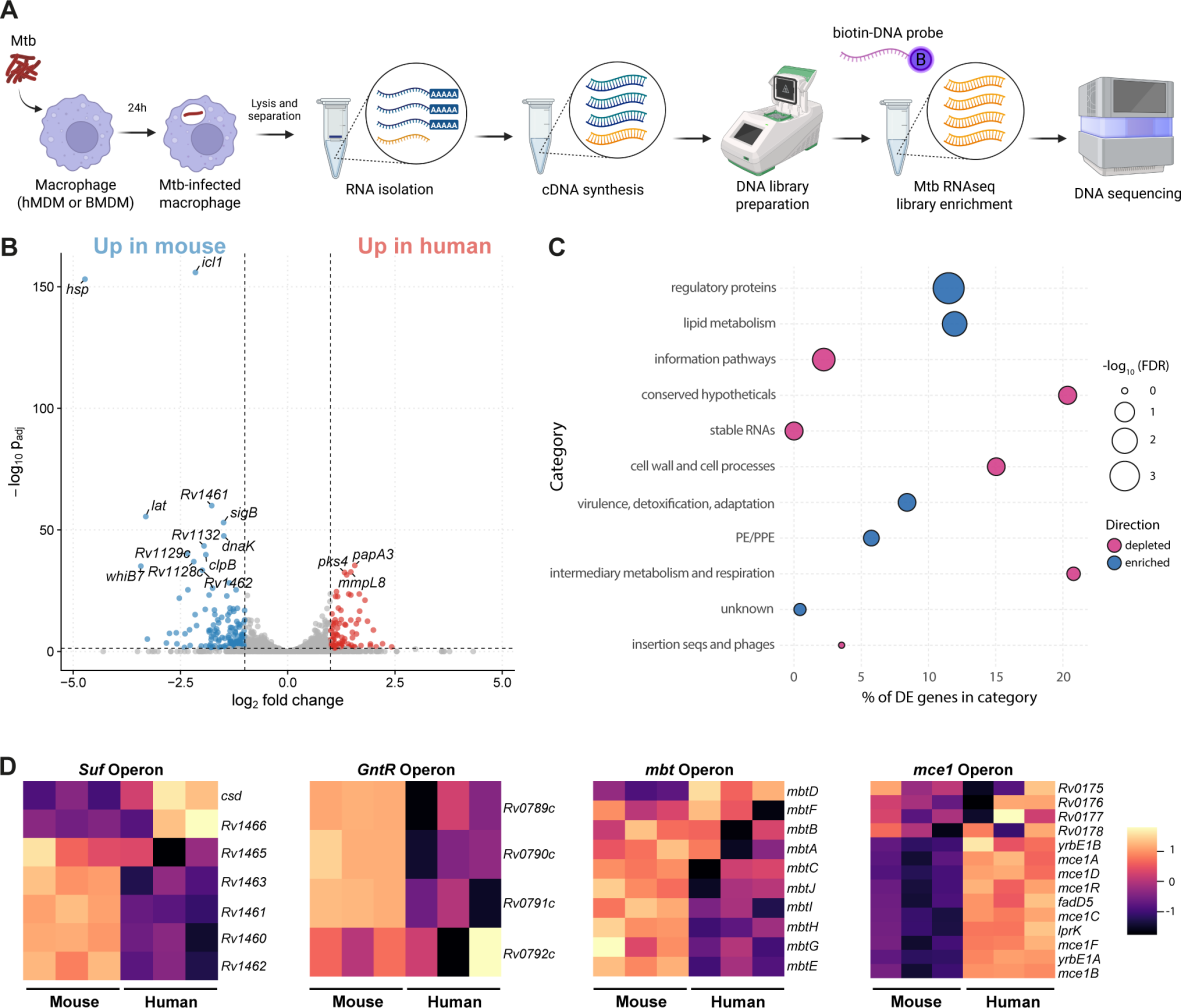
RNA-seq analysis of differentially expressed genes in *M. tuberculosis* (Mtb) during human versus mouse macrophage infection. (**A**) Schematic of intracellular bacterial RNA sequencing workflow. Primary human monocyte-derived macrophages (hMDMs) and mouse bone marrow-derived macrophages (BMDMs) were infected with Mtb H37Rv wild type for 24 h. Cells were lysed, and bacteria and macrophage contents were separated by differential centrifugation and then combined for RNA isolation. Afterwards, cDNA was synthesized and amplified. Hybrid selection was done to enrich bacterial transcripts by using biotinylated-DNA probes complementary to the Mtb H37Rv transcriptome, excluding ribosomal and transfer RNA, before performing sequencing. (**B**) Volcano plot showing differentially expressed genes (DEGs) in Mtb during infection of human versus mouse macrophages. (**C**) Pathway enrichment analysis of Mtb DEGs grouped by functional category, depicting enriched and depleted genes during macrophage infection. (**D**) Heat maps showing Mtb operons differentially expressed during infection of mouse and human macrophages, including Fe–S complex assembly (*Suf* operon), reactive oxygen species response (*GntR* operon), mycobactin synthesis (*Mbt* operon), and fatty acid transport (*Mce1* operon).

We grew Mtb H37Rv wild type in 7H9 medium supplemented with glycerol, Tween, and oleic acid, bovine albumin, dextrose, and catalase (OADC). A single Mtb culture was split and used to infect either murine bone marrow-derived macrophages (BMDMs) or human monocyte-derived macrophages (hMDMs), and RNA was harvested at 24 h post-infection (see Materials and Methods). Because bacterial transcripts are a minor fraction of total RNA in infected cells, we employed an optimized targeted-capture protocol to enrich for Mtb transcripts prior to sequencing (Fig. 1A) (15). We observed that ~20% of Mtb genes were differentially expressed (DE) (*p*_adj_ < 0.05) between mouse and human macrophages (Fig. 1B), with approximately equal numbers higher in human versus mouse cells. Genes with higher expression in human cells included *papA3*, *mmpl8*, *pks4*, *esxP*, and *PPE51*; those higher in mouse cells included *icl1*, *hsp*, *lat*, and *whiB7* (Fig. 1B; Table S1).

Pathway-level analysis using *P*_adj_ < 0.05 and |log_2_FC| > 1 indicated enrichment of genes involved in regulation and lipid metabolism among the differentially expressed set (Fig. 1C). These trends were consistent when relaxing the fold change requirement. Enriched regulators included *prpR*, *mce1R*, and *tcrX*, and lipid-associated genes included *pks1–4*, *papA1–4*, and *umaA*. Motivated by these signals, we examined expression of a curated lipid metabolism gene set (fatty acid-responsive genes, transporters, and cholesterol-catabolism genes). We observed elevated expression of stress-response genes such as iron-stress (e.g., *Rv1460–66* and *csd*, which belong to the *Suf* operon), reactive oxygen species response (e.g., *Rv0789c–92c*, included in the *GntR* operon), as well as mycobactin biosynthesis genes (*mbtB*, *mbtD*, *mbtE*, *mbtI*; part of the *Mbt* operon) in mouse macrophages, whereas components associated with fatty acid import (e.g., *YrbE1A*, *YrbE1B*, *mce1A*, and *mce1B*, which belong to the *Mce1* operon) were more highly expressed in human macrophages (Fig. 1D). It is well known that lipids are very important for Mtb survival and replication during host infection and during axenic growth. Mtb can replicate inside lipid-loaded foamy macrophages (16), and possesses the unique capacity to import and utilize host-derived fatty acids and cholesterol to synthesize its complex and lipid-rich cell envelope (17). In fact, host-derived lipids are the primary carbon source for Mtb *in vivo*, which are catabolized to fuel central metabolic pathways required for Mtb’s persistence (18). In the present work, we show that lipid import and metabolism are enriched in Mtb during macrophage infection, and that macrophages from different host species impose distinct microenvironmental pressures that drive Mtb into separable transcriptional states, with prominent differences in metabolic pathways and regulatory circuits.

### *M. tuberculosis* forms intracellular lipid inclusions in resting murine macrophages but not in resting human macrophages

**Fig 2:**
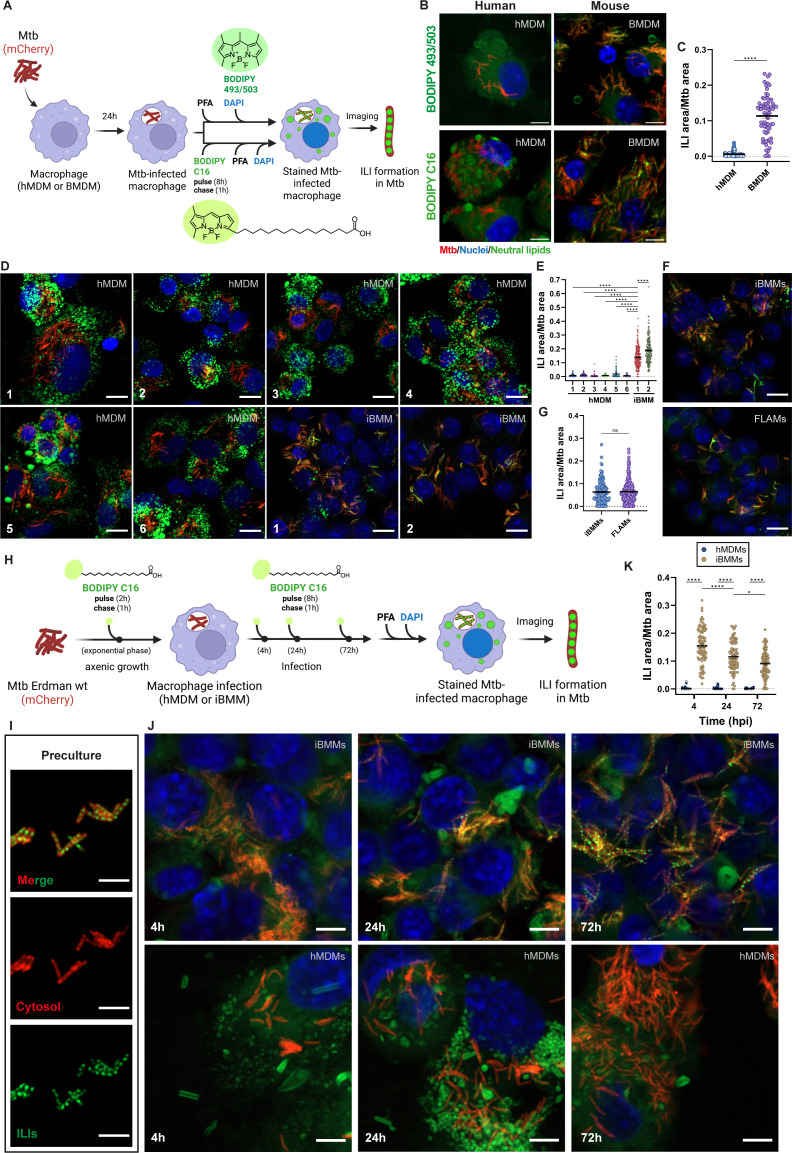
Intracellular lipid inclusions (ILIs) formed by *M. tuberculosis* (Mtb) during macrophage infection are species dependent. (**A**) Primary human macrophages (human monocyte-derived macrophages [hMDM]) and primary mouse macrophages (bone marrow-derived macrophages [BMDMs]) were infected with cytosolic mCherry-expressing Mtb Erdman wild type (wt) for 24 h. In one condition, samples were fixed with paraformaldehyde (PFA) and stained with DAPI (nuclei), and neutral lipids were labeled with BODIPY 493/503. In the other, neutral lipids were labeled by an 8 h pulse with fluorescent palmitate (BODIPY C16) followed by a 1 h chase. Samples were imaged by Airyscan fluorescence microscopy (**B**). Representative Airyscan images showing mCherry-labeled Mtb, nuclei (blue), and neutral lipids (green). Scale bars, 5 µm. (**C**) BODIPY C16 fluorescence signal from Mtb ILIs contained in each Mtb object (either single bacterium or bacterial cluster) in panel** B** was quantified using Cell Profiler; data are presented as ILI area/Mtb area ± SEM (*n* = 3) (****P < 0.0001; two-way ANOVA with Dunnett’s multiple comparisons test). (**D**) hMDMs were cultured in the following media before infection: (1) RPMI + 10% FBS (R10) + M-CSF, (2) R10 + GM-CSF, (3) RPMI + 10% HS + M-CSF, (4) human plasma-like medium (HPLM) + 10% FBS + M-CSF, (5) HPLM + 10% HS + M-CSF, and (6) DMEM + 10% FBS + M-CSF. Immortalized BMDMs (iBMMs) were cultured in (1) DMEM + 10% FBS or (2) R10. Cells were infected for 24 h (MOI = 3 for hMDMs, MOI = 10 for iBMMs), subjected to an 8 h BODIPY C16 pulse and 1 h chase, fixed, stained with DAPI, and imaged by Airyscan fluorescence microscopy. Scale bars, 10 µm. (**E**) Quantification of ILI fluorescence from panel **D**; data represent ILI area/Mtb area ± SEM (*n* = 3) (****P < 0.0001; two-way ANOVA with Tukey’s multiple comparisons test). (**F**) Murine immortalized macrophages (iBMMs) and fetal liver-derived alveolar macrophages (FLAMs) were infected with cytosolic mCherry-expressing Mtb Erdman wt for 24 h, labeled with BODIPY C16 (8 h pulse, 1 h chase), fixed, and imaged as above. Scale bars, 10 µm. (**G**) Quantification of ILI fluorescence from panel **F**; data represent ILI area/Mtb area ± SEM (*n* = 3) (ns [not significant]; unpaired *t* test with Welch’s correction). (**H**) Cytosolic mCherry-expressing Mtb Erdman wt was cultured in 7H9^OADC^ and, at the exponential phase, labeled with BODIPY C16 (2 h pulse, 1 h chase). An unlabeled aliquot of the same pre-culture was used to infect iBMMs or hMDMs. Infections were analyzed at 4, 24, and 72 h post-infection using an 8 h BODIPY C16 pulse and 1 h chase. (**I**) Airyscan image of Mtb pre-culture showing cytosolic mCherry and neutral lipids (green). Scale bars, 2 µm. (**J**) Airyscan images of Mtb-infected iBMMs (top) or hMDMs (bottom) showing mCherry, nuclei (blue), and neutral lipids (green). Scale bars, 5 µm. (**K**) Quantification of ILI fluorescence from panel **J**; data represent ILI area/Mtb area ± SEM (*n* = 3) (*P = 0.0149, ****P < 0.0001; two-way ANOVA with Tukey’s multiple comparisons test). For hMDM data, individual colors denote independent donors. All data represent a minimum of three independent experiments.

We sought to orthogonally validate the transcriptomic differences between human and mouse macrophages with a focus on the lipid metabolism signal. As a high-throughput means to monitor lipid storage, we used a previously established lipid labeling protocol in which neutral lipids are labeled with BODIPY 493/503 (Fig. 2A). BODIPY 493/503 is a lipophilic dye that partitions into neutral lipids such as TAG and cholesteryl esters. Earlier studies using this protocol initially utilized Mtb Erdman, so we first infected primary hMDM or BMDM with Erdman that constitutively expresses a cytosolic mCherry for 24 h. Then, cells were fixed, stained with BODIPY 493/503, and imaged by confocal microscopy (Fig. 2B). Strikingly, BODIPY-stained puncta were observed in the cytosol of intracellular Mtb during infection of mouse macrophages, but these were not present when infecting human macrophages (Fig. 2B). Mtb can import neutral lipids from the host and accumulate them in cytosolic LDs, best known as ILIs (6). Similar to eukaryotic LDs, mycobacterial ILIs also contain a neutral lipid core composed primarily of TAGs surrounded by a single phospholipid monolayer (7). Accumulation of ILIs in Mtb leads to inhibition of bacterial replication and transition to a non-replicating persistence state, and more importantly, ILIs may contribute to the survival, re-activation, and/or transmission of these bacteria following infection (6, 7). We next used an alternative protocol employing BODIPY palmitate (C16), a fluorescent fatty acid analog that can be utilized to dynamically track lipid trafficking to Mtb (19, 20). In this experiment, mCherry-expressing Mtb was used to infect macrophages. After specific infection periods, BODIPY C16 is added to culture media for a defined period of time (pulse) and followed by a 1 h incubation without BODIPY C16 (chase). Using BODIPY C16 as a probe of dynamic lipid trafficking, we observed ILIs in BMDM but not in hMDMs (Fig. 2B and C). These species differences extended to murine macrophage cell lines, including immortalized BMDMs (iBMMs) and RAW264.7 cells, which both displayed ILIs (Fig. S1). We therefore used iBMMs going forward to take advantage of an existing panel of macrophage mutants. As BODIPY C16 facilitated better ILI visualization than BODIPY 493/503, we opted to use BODIPY C16 for the remainder of our experiments. Taken together, these experiments provide visual validation that differential expression of lipid metabolic genes during murine and human macrophage infection leads to changes in lipid handling in Mtb.

We next performed additional experiments to confirm that the differences in ILI formation observed were genuine and not artifacts of media composition. Murine macrophages are commonly cultured in a media base of Dulbecco’s modified Eagle medium (DMEM), while human macrophages are commonly cultured in Roswell Park Memorial Institute (RPMI) 1640 media. Alternative culture media such as human plasma-like media (HPLMs) can be used. Human macrophage culture media are often supplemented with growth factors such as M-CSF or GM-CSF to support differentiation. Furthermore, as an alternative to the use of heat-inactivated fetal bovine serum (FBS) in culture media, heat-inactivated human serum (HS) can be used. To test the hypothesis that the differences in ILI detection in human versus mouse macrophages are attributable to species differences and not protocol differences, human macrophages were cultured in the following media: (i) RPMI + 10% FBS (R10) + M-CSF, (ii) R10 + GM-CSF, (iii) RPMI + 10% HS + M-CSF, (iv) HPLM + 10% FBS + M-CSF, (v) HPLM + 10% HS + M-CSF, and (vi) DMEM + 10% FBS + M-CSF prior to ILI analysis. Conversely, iBMMs were cultured in either (i) DMEM + 10% FBS or (ii) R10. We next repeated our BODIPY C16 pulse-chase experiments and were able to detect ILIs in iBMMs under all culture conditions. ILIs remained undetectable in hMDMs in all culture conditions (Fig. 2D and E). These results further confirmed the differences in Mtb metabolic state between human and murine macrophages.

To determine whether the ILI phenomenon observed in murine macrophages extended to macrophages from different ontogenies, we evaluated murine fetal liver-derived alveolar macrophages (FLAMs) as a distinct lineage model. We compared iBMMs and FLAMs following infection with Mtb and performed the BODIPY C16 pulse-chase experiment. We readily detected ILIs in both iBMMs and FLAMs (Fig. 2F and G).

It is possible that the observed species difference is due to distinct kinetics in ILI formation. To test this, we determined how soon after Mtb infection this differential ILI phenotype is observed. We grew Mtb Erdman expressing cytosolic mCherry in 7H9 medium supplemented with glycerol, Tween, and OADC. At the exponential phase, the capacity for ILI formation was tested by a 2 h BODIPY C16 pulse-1 h chase. This same unlabeled Mtb culture was used to infect either hMDMs or iBMMs. After 4, 24, or 72 h of infection, BODIPY C16 pulse-chase experiments were performed (Fig. 2H). ILIs were observed in the Mtb pre-culture during axenic growth (Fig. 2I). This ILI-competent trait in Mtb was conserved throughout axenic growth in complex media (Fig. S2). Surprisingly, immediately upon hMDM infection, Mtb was devoid of ILIs, and the capacity for ILI formation was not restored at the later infection timepoints. In contrast, during iBMM infection, despite being dimmer right after infection, ILIs were readily formed throughout the course of the infection (Fig. 2J and K). Taken together, these results indicate that access to lipids by intracellular Mtb for ILI formation is different in these two species.

### Knockout of host factors with well-characterized species differences does not affect ILI formation in *M. tuberculosis* during macrophage infection

**Fig 3:**
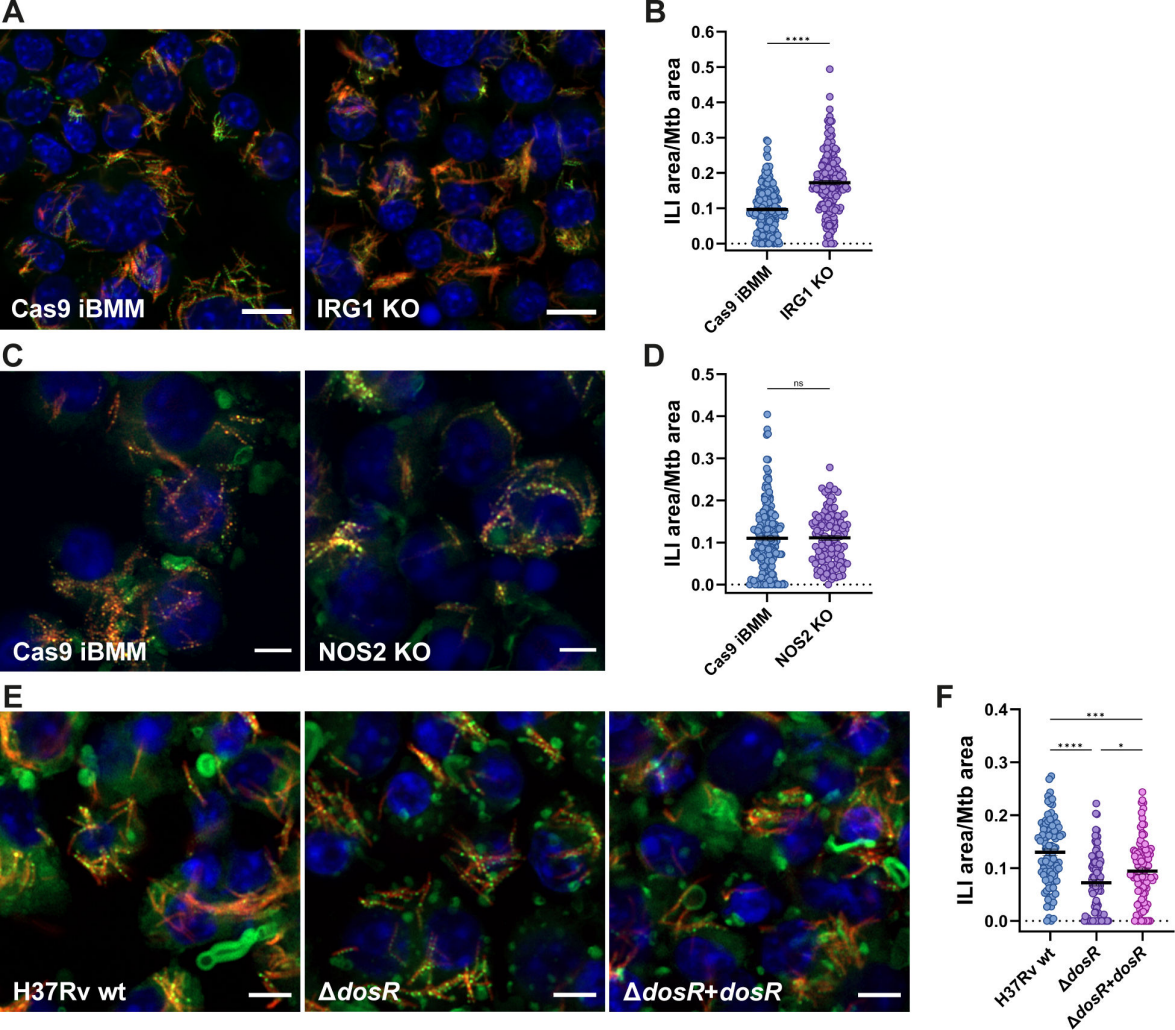
Knockout of mammalian and bacterial factors has different effects on ILI formation in *M. tuberculosis* in macrophages. (**A–D**) Immortalized murine bone marrow-derived macrophages (iBMMs) expressing Cas9, or knockout (KO) mutants for *irg1* (**A**) and *nos2* (**C**) were infected for 24 h with cytosolic mCherry-expressing Mtb Erdman. Following infection, neutral lipids were labeled with BODIPY C16 (8 h pulse, 1 h chase); cells were fixed; nuclei were stained with DAPI; and samples were imaged using Airyscan fluorescence microscopy (**A and C**). (**B and D**) Quantification of ILI signal from panels **A and C** using CellProfiler. Data are shown as ILI area/Mtb area ± SEM (*n* = 3) (ns [not significant], ****P < 0.0001; unpaired t test with Welch’s correction). (**E and F**) iBMMs were infected with Mtb H37Rv wild type (wt), Δ*dosR*, or complemented Δ*dosR + dosR* strains for 24 h; BODIPY C16 pulse-chase was performed, and samples were treated and imaged as above (**E**). (**F**) Quantification of ILI signal from panel **E** using CellProfiler. Data are shown as ILI area / Mtb area ± SEM (*n* = 3) (*P = 0.0173, ***P = 0.0008, ****P < 0.0001; two-way ANOVA with Tukey’s multiple comparisons test). Infections were conducted at an MOI = 10. Scale bars, 5 µm.

Previous studies have identified many host factors that are differentially regulated in human versus mouse macrophages. We therefore tested if these factors accounted for the observed ILI formation in murine versus human macrophages (8, 11, 21, 22). We focused on nitric oxide and itaconate as candidate molecules. The field continues to debate the role of nitric oxide synthase 2 (NOS2) in antimicrobial programs in human cells, though recent studies suggest that the genomic architecture of the human NOS2 locus contributes to distinct expression patterns between humans and mice (8, 9). The enzyme responsible for itaconate production, *cis*-aconitate decarboxylase 1, encoded by the gene *irg1*, has a point mutation in humans that reduces itaconate production by orders of magnitude compared to the murine enzyme (3, 10, 23). Both nitrosative species and itaconate have been implicated in the regulation of Mtb metabolism (1, 2, 4, 9, 24). We used CRISPR-Cas9 to knockout *irg1* or *nos2* in murine macrophages. After confirming knockout by Western blotting (Fig. S3), we again performed our BODIPY C16 pulse-chase experiments in these macrophages infected with Mtb Erdman (Fig. 3). Neither deletion of *irg1* (Fig. 3A and B) nor *nos2* (Fig. 3C and D) was able to disrupt ILI formation.

### Deletion of bacterial genes involved in dormancy or virulence has different effects on ILI formation

Previous studies have argued that ILI formation in axenic culture occurs in response to hypoxia or other stresses associated with dormancy. The transcriptional regulator DosR has been implicated in the regulation of these stress responses (25, 26). We obtained an Mtb mutant lacking *dosR*, as well as its complemented strain, and generated fluorescent versions of both (26). We next infected iBMMs with these strains and performed our BODIPY C16 pulse-chase experiments. Deletion of *dosR* did not disrupt ILI formation in Mtb during murine macrophage infection, consistent with previous predictions by others (27) (Fig. 3E and F). In our experiments, BODIPY C16 was added extracellularly and thus must be taken up by the macrophage and then trafficked to the intracellular bacterium. We hypothesized that Mtb mutants that influence phagosome integrity might impact BODIPY C16 acquisition during infection. We generated a fluorescent EccCa1 transposon mutant (*eccCa1:Tn*) and next performed our BODIPY C16 pulse-chase experiments. EccCa1 is an ATPase that regulates activity of the ESX-1 secretion system (28). The ESX-1 system exports proteins that contribute to phagosomal membrane damage (29). Strikingly, during iBMM infection, the *eccCa1:Tn* mutant lacked detectable ILIs (Fig. S4A and B). We next asked whether loss of ESX-1 activity disrupted BODIPY C16 acquisition during axenic growth, to assess whether the phenotype was specific to macrophage infection or it might reflect a general defect in lipid import. In contrast to the loss of ILI signal in iBMMs observed with the ESX-1 mutant, ILIs were still observed in the ESX-1 mutant in axenic culture (Fig. S4C). This suggests that the ESX-1 system is required for ILI formation by the bacteria during macrophage infection, in addition to its canonical roles in phagosomal membrane disruption and virulence effector secretion.

### Impaired triacylglycerol synthesis in human macrophages permits ILI formation in infecting *M. tuberculosis*

**Fig 4:**
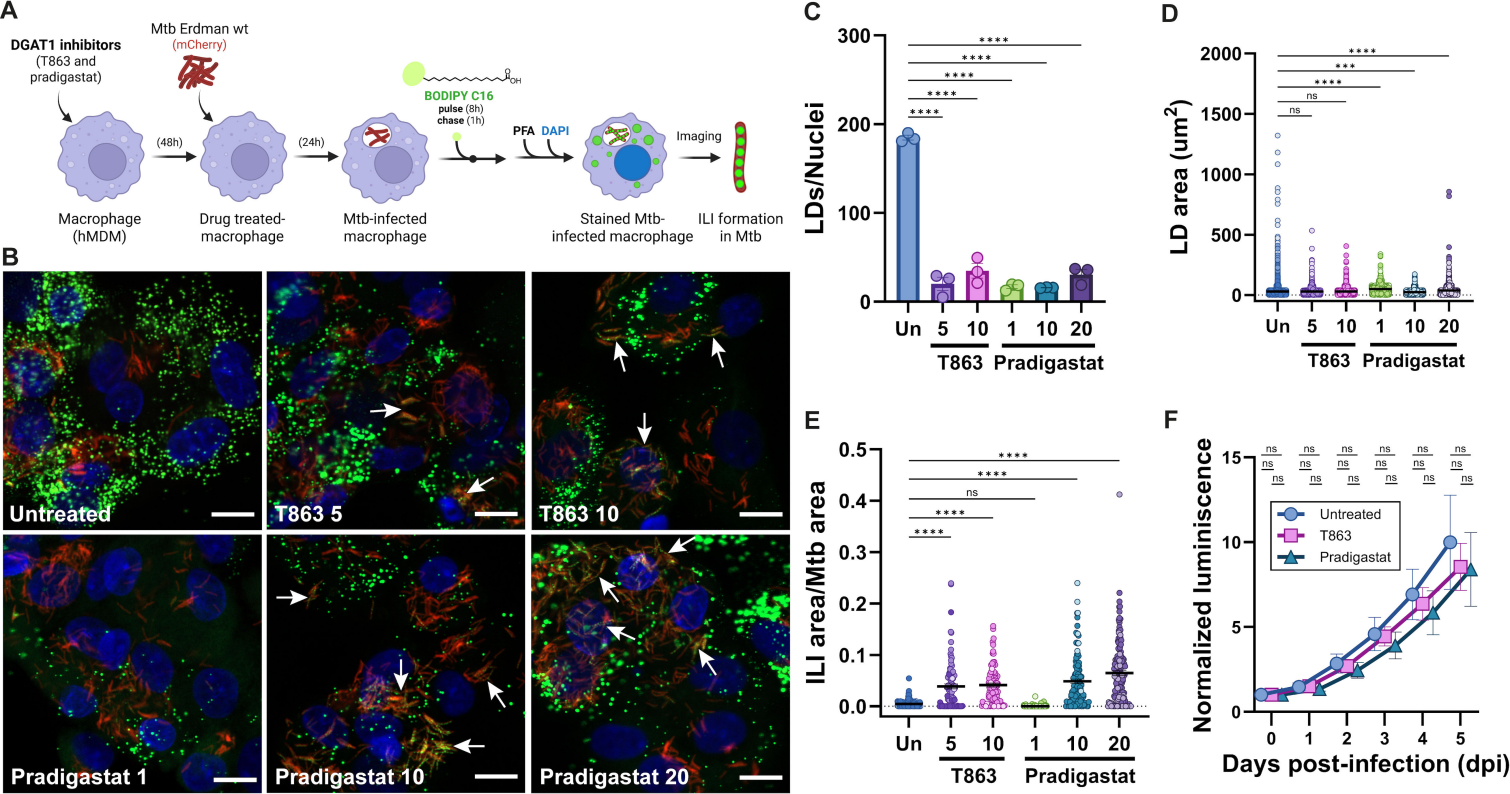
Inhibiting host TAG synthesis with diacylglycerol acyltransferase 1 (DGAT1) inhibitors permits intracellular lipid inclusion formation in Mtb in human macrophages. (**A and B**) Human macrophages (hMDMs) were pre-treated for 48 h with two distinct DGAT1 inhibitors—T863 (5 or 10 mg/L) or pradigastat (1, 10, or 20 mg/L)—and then infected for 24 h with cytosolic mCherry-expressing Mtb Erdman at an MOI = 3. Following infection, neutral lipids were labeled with BODIPY C16 (8 h pulse, 1 h chase); cells were fixed; nuclei were stained with DAPI; and samples were imaged using Airyscan fluorescence microscopy. White arrows indicate Mtb containing ILIs. Scale bars, 10 µm. (**C–E**) Quantification of (**C**) lipid droplet (LD) number per nucleus, (**D**) LD area (µm²), and (**E**) ILI area per Mtb area from images in panel **B**. Data are presented as mean ± SEM (*n* = 3) (**C**), as LD area ± SEM (*n* = 3) (**D**), or as ILI area/Mtb area ± SEM (*n* = 3) (**E**). Points are color-coded to denote individual human donors. (**F**) For bacterial burden measurement, hMDMs were pre-treated for 48 h with either T863 or pradigastat (10 mg/L) and then infected with luxABCDE cassette-expressing Mtb H37Rv at an MOI = 3. Following infection, luminescence was measured after 0–5 days post-infection (dpi). Bacterial burden is represented as normalized luminescence (fold change relative to dpi = 0) for each sample ± SEM (*n* = 3) (ns. not significant; *** *P* = 0.0006, ****P < 0.0001; two-way ANOVA with Dunnett’s multiple comparisons test).

While our experiments with Mtb mutants expanded our understanding of bacterial factors influencing lipid acquisition in murine macrophages, the factors contributing to the observed interspecies differences in Mtb ILI formation remained unclear. Our data did not indicate a defect in BODIPY C16 import by hMDMs, as host LDs were readily loaded with BODIPY C16 (Fig. 2). The rate-limiting enzyme involved in TAG synthesis in mammalian cells is diacylglycerol acyltransferase 1 (DGAT1), which catalyzes the synthesis of TAG from diacylglycerol and long-chain fatty acyl-CoAs (30). To examine the role of TAG synthesis and host LD formation in Mtb ILI formation, human macrophages were pre-treated with two DGAT1 inhibitors (T863 and Pradigastat) following a previously established protocol linking LD formation to the efficacy of the lipophilic antibiotic bedaquiline (31). hMDMs were pre-treated for 48 h with vehicle or drug prior to infection with mCherry Mtb (Fig. 4A). Cells were infected with Mtb in the absence of extracellular drug for 4 h. After washing away extracellular Mtb, the medium containing vehicle or drug was added back for the remainder of the experiment. Twenty-four hours following infection, we performed our BODIPY C16 pulse-chase experiment and visualized samples by fluorescence microscopy (Fig. 4B). We first confirmed that drug treatment decreased the formation of host LDs by confocal microscopy (Fig. 4C and D). We then quantified ILI formation as a function of drug and concentration and found that inhibition of host TAG synthesis resulted in a detectable ILI signal in Mtb (Fig. 4E). The enhanced Mtb ILI signal in hMDMs was dose dependent and occurred with both drugs. Notably, this inhibition did not result in detectable ILI signal in all intracellular Mtb, suggesting that host TAG synthesis may be one component of the regulation of Mtb metabolic state. We next tested whether modulating Mtb ILI formation in hMDMs through inhibition of TAG synthesis could affect bacterial burden. T863 or pradigastat-pre-treated hMDMs were infected with lux-expressing Mtb H37Rv, and bacterial luminescence was measured as a proxy for bacterial load. However, we observed no significant change (Fig. 4F). Similarly, human THP-1 macrophage knockdown of DGAT1 or perilipin 2 (PLIN2), genes involved in lipid droplet biology, showed reduced formation of host LDs (Fig. S5A through C; Fig. S6), enhanced Mtb ILI formation (Fig. S5D; Fig. S6), and unaltered bacterial load (Fig. S5E). These findings suggest that fatty acids stored as TAGs in host LDs are not readily available to Mtb for ILI formation in hMDMs. Instead, host LDs may sequester lipids away from the bacteria, or imported lipids may be preferentially used for cell wall synthesis required for bacterial elongation and replication.

## DISCUSSION

As an intracellular pathogen, Mtb resides primarily within macrophages, where it must adapt to host-imposed metabolic and immune constraints. Our cross-species analyses reveal that these pressures differ markedly between mouse and human macrophages, driving Mtb into distinct physiological states. In mouse macrophages, Mtb upregulates iron acquisition, oxidative stress, and lipid metabolism pathways, including the *suf* and *mbt* operons, whereas in human macrophages, the bacterium preferentially induces fatty acid import and beta-oxidation machinery, such as *mce1*, *fadD*, and *yrbE* genes (Fig. 1). These transcriptional differences manifest in a striking phenotypic divergence: Mtb forms ILIs during infection of mouse macrophages but not in human macrophages (Fig. 2; Fig. S1).

ILIs have been observed previously in sputum isolates and in macrophages under specific stress conditions (e.g., reference 32), but their biological origin and regulation have remained incompletely understood. Our data reveal that ILI formation is not an invariant feature of Mtb infection but rather a host species-specific outcome reflecting distinct intracellular environments. Multiple stressors such as hypoxia, nutrient limitation, or redox stress can promote TAG accumulation in Mtb (6, 16, 33, 34), yet our findings demonstrate that even in the absence of such stimuli, infection of mouse macrophages alone is sufficient to trigger ILI formation. Mtb ILI formation has been observed previously during infection of murine macrophages (32). Also, ILI formation in Mtb was shown to be abolished by IFN-γ-treatment of the macrophages, displaying an opposite relationship with the presence of LDs in the host (32). Furthermore, Mtb ILI formation was not observed during infection of human macrophages, and treatment with IFN-γ did not provoke ILI formation in Mtb (32). In the present work, we corroborated these observations, and demonstrated that human macrophages do not trigger ILI formation in Mtb, underscoring that Mtb’s metabolic state is tightly coupled to host-specific cues (Fig. 2; Fig. S1). ILI formation is also observed in Mtb during axenic growth in complex media (Fig. S2).

Mtb possesses respiratory flexibility, which allows it to grow optimally in aerobic conditions but also survive under hypoxia, still maintaining bioenergetic homeostasis. Mtb is considered a prototrophic bacterium that possesses a broad metabolic repertoire and a unique lipid-rich and complex cell envelope that provides the pathogen the ability to survive within diverse microenvironments during host cell infection (35). The Mtb cell envelope represents a highly hydrophobic and multilayered dynamic barrier whose biophysical structural properties may also impact host infection (36). The ancient co-evolution of Mtb with its hosts has imposed selective pressures that have shaped and continue to influence its physiology and metabolism (37, 38). Mtb’s ability to co-catabolize both carbon and nitrogen sources, as well as to modify its responses to different host cell types, magnifies its metabolic flexibility and augments its success as a pathogen (39). Taken together, Mtb’s transcriptional plasticity allows it to respond to diverse host environmental cues, and it denotes that the bacterial metabolic state is a major driver of its physiology and resistance to the host antimicrobial responses.

In the present study, we found that ILI formation in Mtb does not depend on host nitric oxide or itaconate production (Fig. 3), two antimicrobial metabolites that differ between mouse and human macrophages, suggesting that other, as yet undefined host signals govern this process. Instead, our data implicate host neutral lipid metabolism as a key determinant. Inhibition of host TAG synthesis through DGAT1 blockade or knockout unexpectedly enabled Mtb to access host lipids and restored ILI formation (Fig. 4; Fig. S5), suggesting that sequestration of fatty acids within host LDs can modulate bacterial lipid metabolism. This observation highlights an underappreciated connection between host LD homeostasis and bacterial nutrient availability. It also suggests that the interplay between host TAG metabolism and bacterial lipid storage could influence the intracellular efficacy of lipophilic antimicrobials, as previously proposed (31).

We also identified bacterial determinants of ILI formation. While the DosR regulon, long associated with the hypoxia response (25, 26), was dispensable (Fig. 3), the ESX-1 secretion system (29) was required. Mtb mutants lacking ESX-1 failed to form ILIs and exhibited restricted survival and replication within macrophages (Fig. S4). These findings might extend the role of ESX-1 beyond virulence and phagosomal rupture to include access to host-derived lipids; however, we and others have been unable to complement the mutation to demonstrate reversibility of the phenotype. Future studies should test if mutating other ESX-1 factors, such as espACD operon or CFP10/ESAT6 (29), that could be complemented affects ILI formation in intracellular Mtb. Also, it would be interesting to see, in addition to ESX-1, if mutating other secretion systems that have been shown to be required for permeabilization of the phagosomal membrane secretion, such as ESX-2 and ESX-4 (40, 41), would also affect Mtb ILI formation during macrophage infection. Nevertheless, lipid storage and translocation to intracellular Mtb seems to be affected during human macrophage infection (Fig. 2), and the upregulation in the mce1 gene cluster (Fig. 1) may be a compensatory response in an attempt by Mtb to import more fatty acids. Consistent with this, a mutant that does not import fatty acids highly upregulates the mce1 gene cluster during infection in murine macrophages (42). We speculate that species-specific differences in ESX-1 activity or the degree of phagosomal permeabilization may contribute to the divergent lipid phenotypes observed in human and mouse macrophages.

During infection, Mtb relies primarily on host-derived lipids as carbon sources for energy production and to synthesize its own cell envelope. Formation of ILIs as a TAG storage bacterial organelle was first observed by Waltermann et al. (43, 44). Although TAG deposition within LDs is conserved in eukaryotic cells from plants, animals, and yeast, ILI formation has only been observed in a small group of prokaryotes, including actinomycetes such as *Mycobacterium*, *Rhodococcus*, *Nocardia*, and *Streptomyces* (45). Host fatty acid uptake in gram-positive pathogenic bacteria, such as *Streptococcus pneumoniae* and *Staphylococcus aureus*, is essential for membrane phospholipid synthesis. Also, in gram-negative human pathogens, such as *Salmonella typhimurium*, *Pseudomonas aeruginosa*, *Acinetobacter baumannii*, and *Escherichia coli*, the utilization of host-derived fatty acids is key for bacterial virulence (45). Given that *de novo* fatty acid synthesis by fatty acid synthase II is highly energetically demanding, some pathogenic bacteria have evolved to use exogenously acquired fatty acids only for membrane biogenesis and replication. Apart from *M. tuberculosis* and *A. baumannii*, it would be interesting to identify other pathogens that similarly depend on host fatty acids during infection and to determine how host-derived cues modulate their metabolic states and promote storage of TAGs or other lipids within ILIs.

Collectively, our findings uncover a new axis of host–pathogen metabolic interaction in which ILI formation serves as a readout of Mtb’s access to host lipids. This process seems to depend on host TAG synthesis and varies fundamentally between macrophage species. While ILIs have traditionally been viewed as markers of dormancy, our results suggest they can also arise as a context-dependent adaptation to the metabolic landscape of the host cell. Future work should elucidate the molecular signals that trigger ILI formation in mouse macrophages, elucidate if other human macrophage types (e.g., tissue-resident alveolar macrophages) restrict ILI formation in intracellular Mtb, define whether similar conditions occur *in vivo*, and determine how modulation of host lipid metabolism or ESX-1 activity reshapes bacterial physiology. Understanding these species-specific interfaces between host and pathogen metabolism will be critical for identifying new therapeutic strategies to restrict Mtb survival in human macrophages.

## MATERIALS AND METHODS

### Bacteria and media

Mtb H37Rv and Erdman strains were grown at 37°C in 7H9^OADC^ medium (Middlebrook 7H9 Broth supplemented with 0.2% glycerol, 0.05% Tween-80, and OADC) with shaking at 65 rpm. Antibiotics were added to the media when required, at a concentration of 20 µg/mL Zeocin (Thermo Fisher Scientific), for pBB84 plasmid-containing strains expressing a cytosolic mCherry. All Mtb strains utilized in this work are PDIM positive.

### BODIPY stock preparation

For neutral lipid labeling during either bacterial axenic growth or during macrophage infection, a 5 mM stock of BODIPY palmitate (C16) (BODIPY FL C_16_, Thermo) was prepared in dimethyl sulfoxide (DMSO). The 5 mM BODIPY C16 stock was diluted in 1% fatty acid-free BSA prepared in phosphate-buffered saline (PBS) solution to obtain a 100 µM BODIPY C16 stock, as previously described (19). The 100 µM BODIPY C16 stock was vortexed until the solution turned green and kept at 37°C wrapped in aluminum foil to protect it from light. For lipid incorporation analysis during axenic culture, at the time of labeling, the 100 µM BODIPY C16 stock was diluted in 7H9^OADC^ and vortexed to obtain an 8 µM working solution. For lipid incorporation analysis during macrophage infection, the 100 µM BODIPY C16 stock was diluted in the desired media to obtain an 8 µM BODIPY C16 working solution in media. Alternatively, a 5 mM stock of BODIPY 493/503 (Thermo) was prepared in DMSO and further diluted in PBS to obtain a working solution at 5 µM, which was used for neutral lipid staining post-fixation.

### BODIPY C16 lipid labeling during axenic growth

Mtb strains were grown in 7H9^OADC^ until the logarithmic phase (OD ≤ 1) and a 0.5 mL aliquot was transferred to a 1.5 mL microcentrifuge tube. Samples were centrifuged at 3,900 rpm for 5 min at room temperature. Then, the supernatant was removed, and the pellet was re-suspended in 0.5 mL of 8 µM BODIPY C16 working solution in 7H9^OADC^. Samples were protected from light by wrapping the tube in foil, and they were incubated at 37°C for 2 h with shaking (pulse). After the pulse, samples were centrifuged at 3,900 rpm for 5 min at room temperature. The supernatant was removed, and the pellet was re-suspended in 0.5 mL of 7H9^OADC^ and incubated again at 37°C for 1 h with shaking (chase). After the chase, samples were centrifuged at 3,900 rpm for 5 min at room temperature; supernatant was removed; and samples were fixed by re-suspending the pellet in 0.5 mL of 4% paraformaldehyde (PFA) in PBS. After fixation, two series of washing steps with 0.5 mL of PBS were done, and samples were re-suspended in Milli-Q water, plated, and visualized by high-resolution fluorescence microscopy.

### Human macrophage differentiation and culture

For human cell isolation and differentiation, de-identified, truly anonymous buffy coats were obtained from the Massachusetts General Brigham Hospital. Peripheral blood mononuclear cells were isolated by density-based centrifugation with Ficoll (GE Healthcare). CD14+ monocytes were further isolated using a CD14+ selection kit (Stemcell). Isolated monocytes were differentiated to macrophages using a variety of medium conditions:

1. Buffy media (phenol-free RPMI 1640 [Gibco] supplemented with 10% heat-inactivated fetal-bovine serum [HI-FBS] [Gibco], 10 mM HEPES [Corning], and 2 mM L-glutamine [Sigma], along with 25 ng/mL of either M-CSF or GM-CSF [BioLegend], depending on the experimental design).
2. Human Plasma Like Media (HPLM media; Invitrogen) supplemented with 10% HI-FBS [Gibco], 10 mM HEPES [Corning], and 2 mM L-glutamine [Sigma], along with 25 ng/mL of M-CSF [BioLegend]).
3. BMDM media (DMEM [Gibco] supplemented with 10% HI-FBS [Gibco], 10 mM HEPES [Corning], and 2 mM L-glutamine [Sigma], along with 25 ng/mL of M-CSF [BioLegend]).

In some cases, heat-inactivated human AB serum was used instead.

Cell differentiation was carried out by plating the cells on ultra-low-attachment plates or in 12-well chamber slides (IBIDI). In the case of cells plated in ultra-low-attachment plates, after 6 days of differentiation, cells were detached using PBS supplemented with 4 mM EDTA and were replated in 12-well chamber slides at a cell density of 2.5 × 10^5^ cells/well. The cells were allowed to re-adhere overnight prior to infection.

### BMDM isolation and differentiation

Femurs and tibias were harvested from C57BL/6 mice, stripped of muscle, and washed in ethanol and sterile PBS. Under sterile conditions, the ends of the bones were cut, and marrow was flushed into DMEM (Gibco) containing 10% HI-FBS (Bio-Techne) and 1% penicillin-streptomycin (Gibco) using a 27 G needle and syringe. A single-cell suspension was achieved by repeatedly passing the marrow through the needle and filtering it through a pre-wetted 70 µm filter. Cells were pelleted and re-suspended in 2 mL ACK (ammonium-chloride-potassium) lysis buffer (Gibco) for 1 min on ice to lyse red blood cells. Following red blood cell lysis, cells were re-suspended at 1.5 × 10^6^ cells/mL in DMEM (Gibco) containing 10% HI-FBS (Bio-Techne), 1% penicillin-streptomycin (Gibco), and 25 ng/mL murine M-CSF (PeproTech). Two milliliters (3 × 10^6^ cells) was plated per well on a six-well, tissue culture-treated dish. A 50% medium replacement was performed at days 2 and 4 post-plating. Prior to Mtb infection, macrophages were washed with antibiotic-free media, and all infections and post-infection experiments were performed in the absence of antibiotics.

### Murine macrophage cell line culture (iBMM, RAW, and FLAMs)

For other murine macrophages such as iBMMs and RAW264.7 macrophages (RAWs), D10 (DMEM supplemented with 10% HI-FBS and 2 mM L-glutamine) media were used. For the culture of FLAMs (46) supplemented with 10% HI-FBS, 30 ng/mL recombinant mouse GM-CSF and 20 ng/mL recombinant human TGF-β1 were used. In all cases, murine macrophages were plated at a cell density of 1.75 × 10^5^ cells/well in 12-well chamber slides. NOS2 and IRG1 KO lines were generated by electroporation of synthetic sgRNAs into Cas9-expressing iBMDMs using the Neon Transfection System (Thermo Fisher) with the following conditions: 1,400 V, 10 ms, and two pulses. Monoclonal knockout lines were generated by limited dilution plating.

### Preparing *M. tuberculosis* cultures for infection

Mtb strains were grown in 7H9^OADC^ until the logarithmic phase (OD ≤ 1), and for each experiment, a 3–5 mL aliquot was transferred to a 15 mL conical tube. Mtb culture was pelleted at 3,900 rpm for 5 min at room temperature. The supernatant was removed, and the pellet was washed by re-suspending it in an equal volume of PBS and centrifuging at 3,900 rpm for 5 min at room temperature. Then, the supernatant was removed, and large Mtb clumps were allowed to settle down by centrifuging at 500 rpm for 5 min at room temperature. Afterwards, the supernatant was collected, and the OD_600_ was measured using PBS as a blank. Assuming an OD_600_ of 1 = 3e^8^ Mtb/mL, the volume needed for the desired MOI was calculated. hMDMs and mouse macrophages were infected to achieve an infection rate of approximately 50%.

### BODIPY C16 lipid labeling during infection

For hMDM infection, an MOI of 3 was used, and for murine macrophages, an MOI of 10 was used. Mtb phagocytosis proceeded for 4 h at 37°C with 5% CO_2_. After phagocytosis, Mtb-containing media were removed from wells, and two serial washing steps were performed using pre-warmed PBS. Then, infections were incubated at 37°C with 5% CO_2_ for 24–72 h, depending on the experimental design.

To perform the BODIPY pulse-chase analysis, the 100 µM BODIPY C16 stock was diluted with the appropriate media to obtain an 8 µM BODIPY C16 working solution, and it was added to the appropriate wells. Samples were incubated for 8 h at 37°C with 5% CO_2_ (pulse), and then 8 µM BODIPY C16-containing media were removed from the wells and were replaced with media only. Samples were incubated for 1 h at 37°C with 5% CO_2_ (chase). Afterwards, media were removed from the wells, and samples were fixed with 4% PFA in PBS. Then, the samples were washed twice with PBS and stained with DAPI, and chamber slides were mounted and visualized by high-resolution fluorescence microscopy.

### THP-1 cell culture and differentiation

LentiX-HEK293T (Takara) was cultured in complete DMEM (Corning) supplemented with 10% heat-inactivated FBS (Gibco) and maintained under 70% confluency. The monoclonal THP-1 Cas9+ cell line is a gift from Ramnik Xavier and was cultured in RPMI 1640 media (Gibco) supplemented with 10% heat-inactivated FBS, 1% HEPES (Corning), and 1% L-glutamine (Sigma). For differentiation of THP-1, the cells are incubated in complete media supplemented with phorbol 12-myristate 13-acetate (Sigma) at a final concentration of 40 nM for 24 h, then detached using PBS supplemented with 4 mM EDTA (Gibco), seeded in chamber slides (Ibidi) at 2.5 × 10^5^ cells/well, and cultured in fresh complete media for another 24 h before downstream experiments.

### Production of lentiviral vectors

For lentiviral production, 10 cm^2^ plates were seeded with 5.67 × 10^6^ LentiX-HEK293T per plate in 8 mL media 20 h prior to transfection. Lentiviral plasmids pMD2.G (Addgene #12259), psPAX2 (Addgene #12260), and transfer plasmids were transfected at a mass ratio of 1:3:5 with Lipofectamine 3000 (Thermo Fisher Scientific) following the manufacturer’s protocol. The media were replaced 6 h post-transfection. Lentivirus was harvested 48 and 72 h post-transfection, with a full volume media change at the 48 h harvest. Media were collected and centrifuged at 300 × *g* for 5 min at 4°C to discard cell debris, then concentrated 50-fold vol/vol with Lenti-X Concentrator (Takara) following the manufacturer’s protocol. Lentivirus was then stored at −80°C in single-use aliquots.

### Generation of polyclonal THP-1 knockout cells

CROPseq-multi-v2-Puro-BsmBI_entry vector (Addgene # 225752) was a gift from Paul Blainey. The arrayed guide gene block is designed as described (47), with the top two guides ranked by CRISPick (48, 49) and synthesized by Twist Bioscience. Lentiviral vectors were produced as described. THP-1 Cas9+ cells were transduced at high MOI, supplemented with 8 µg/mL polybrene, and spinfected at 800 × *g* over 45 min at 32°C. The cells are then expanded and selected for at least 7 days under 2 µg/mL puromycin before downstream experiments.

### THP-1 macrophage protein isolation and Western blotting

THP-1 cell clones were differentiated as described above and plated to a density of 1 × 10^6^ cells/mL in ultra-low-attachment T-25 plates in duplicate. After 24 h of differentiation, a replicate of the samples was treated with 250 µM oleate/NaOH (Sigma) for 4 h. Then, cells were washed with PBS and lysed in 1× RIPA Triton X-100 (Thermo Scientific) containing 1% Triton X-100 and 0.1% SDS, plus Halt protease inhibitor (Thermo Scientific). Lysates were incubated for 30 min in ice, then cleared by centrifugation at 14,000 × *g* for 15 min at 4°C. Protein concentration in clarified lysates was quantified by the 562 nm Pierce BCA Protein Assay Kit (Thermo Scientific). Each lysate sample was boiled with 1× Laemmli SDS sample buffer (Thermo) for 5 min at 95°C. For each lysate, ~10 µg was loaded per well in a 4%–12% Bis-Tris SDS gel (Invitrogen), then transferred into a nitrocellulose membrane using the iBlot 2 Dry Blotting System (Invitrogen). Membranes were blotted at room temperature for 1 h in Intercept TBS blocking buffer (LICORbio) with shaking and then incubated overnight at 4°C with shaking, with the following primary antibodies: DGAT1 rabbit monoclonal antibody (Cell Signaling Technology, #27469), PLIN2 rabbit monoclonal antibody (Invitrogen, #MA5-51419), beta-actin mouse monoclonal antibody (Santa Cruz Technology, sc-47778), and GAPDH mouse monoclonal antibody (Invitrogen, #MA5-15738). Primary antibodies were used diluted 1:2,000 in Intercept TBS T20 antibody diluent (LICORbio). Primary-stained membranes were washed three times for 5 min each with shaking with Tris-buffered saline with 0.1% Tween-20 (TBS-T) and incubated for 2 h at room temperature with shaking with fluorescent secondary antibodies (LICORbio: IRDye 680RD goat antirabbit IgG and IRDye 800CW goat antimouse IgG) at a 1:5,000 dilution in Intercept TBS T20 antibody diluent (LICORbio). Secondary-stained membranes were washed three times for 5 min each with shaking with TBS-T before imaging in the Licor Odyssey CLx Imager. Representative blots for DGAT1 and PLIN2 are shown in Fig. S6.

### High-resolution fluorescence microscopy

For imaging Mtb grown axenically, PFA-fixed samples were washed twice with PBS and re-suspended in Milli-Q water. Depending on the bacterial density of the samples, appropriate dilutions were prepared in Milli-Q water and samples were applied to 35 mm dishes with 14 mm diameter glass coverslips uncoated (Mattek). After the samples dried at room temperature, another set of dishes was prepared with 1% agarose in Milli-Q to mount the samples.

For imaging the Mtb-infected macrophages in 12-well chamber slides, PFA-fixed samples were washed twice with PBS, and half of each sample was treated with 60% isopropanol to permeabilize the cells. After DAPI staining, samples were washed twice with PBS and mounted using ProLong Diamond Antifade Mountant (Thermo) and VWR Coverslips 24 × 60 mm no. 1.5 (Avantor). Samples were allowed to cure for at least 24 h before imaging. Both plates and chamber slides were imaged using confocal fluorescence microscopy using the Cell Discoverer 7 microscope (Zeiss) and the modular image acquisition software ZEN (Zeiss). The Alexa Fluor 568 (AF568) channel was used to acquire the mCherry signal, and the Alexa Fluor 488 (AF488) channel was used to acquire the green fluorescence from neutral lipids stained with BODIPY C16 or BODIPY 493/503. Images were acquired using the 20× and 40× objective lenses at a resolution of 2,048 × 2,048 pixels. Images were acquired with a 1.0 Airy unit pinhole. Images acquired by confocal or Airyscan microscopy were exported as .czi files and analyzed using ImageJ and Cell Profiler software. AF568-positive primary objects comprising either single or clustered bacterial objects were defined as “Mtb objects,” and AF488-positive secondary objects overlapping Mtb objects were defined as ILIs. A minimum of 500 Mtb objects per donor per condition were quantified.

### RNA extraction and purification

Bacterial RNA was harvested from infected macrophages as previously described (14). Infected macrophages were lysed in TRIzol (Thermo Fisher Scientific) and centrifuged at 10,000 × *g* for 20 min at 4°C to pellet intact bacteria. The supernatant, representing the host RNA fraction, was collected and filtered twice through a 0.2 µm PVDF filter to ensure pathogen inactivation. The bacterial pellet was re-suspended in fresh TRIzol containing Lysing Matrix B beads (MP Biomedicals) and disrupted using a bead beater homogenizer (MP Biomedical) at 6.5 m/s for 45 s, followed by a 5 min incubation on ice and a second 30 s bead-beating cycle. Following lysis, the host and bacterial RNA fractions were combined, then extracted with chloroform (200 µL per 1 mL TRIzol), and purified using on-column cleanup with DNase treatment (RNA Clean & Concentrate, Zymo Research).

### Library preparation and sequencing

Total RNA (200 ng) was used as input for strand-specific library preparation with the Agilent SureSelect XT HS2 RNA Kit, following the manufacturer’s instructions, with a 1:2 adapter dilution and an additional 0.8×/1.2× double SPRI post-ligation to deplete ribosomal RNA. For intracellular bacterial RNA sequencing, 200 ng unpooled libraries were hybrid-selected for bacterial transcripts using a custom set of biotinylated probes complementary to the Mtb H37Rv transcriptome, excluding ribosomal and transfer RNAs, and probes were used at a 1:5 dilution to account for bacterial transcripts being a low proportion of input libraries. All libraries were sequenced on an Illumina NextSeq 550 using 150 bp reads.

### Bioinformatic processing

Sequencing reads were processed using a previously published in-house pipeline for intracellular bacterial RNA sequencing (15). Briefly, the nf-core dual RNA-seq pipeline (v1.0.0) was modified to allow for UMI deduplication (umi_tools v1.0.1). UMIs from both mates of each read pair were merged and added to corresponding read headers using umi-tools extract. Reads were aligned using STAR to a composite host–pathogen reference genome generated by dual RNA-seq, comprising either the mouse (GRCm39) or human genome (GRCh38, p13) and Mtb H37Rv (NC_000963). The aligned reads were subsequently indexed with samtools, deduplicated using umi-tools dedup, and quantified with HTSeq.

### RNA sequencing analysis

Differential expression was analyzed using DESeq2, and log_2_ fold changes were adjusted using apeGLM (50). Briefly, statistical significance was determined with a Wald test, and *P* values were adjusted using Benjamini–Hochberg correction. Genes with an adjusted *P* value less than 0.05 and absolute log_2_ fold change greater than 1 were considered significant.

Functional enrichment analysis of Mtb differentially expressed genes was performed using a custom R pipeline implemented in mtb_enrichment.R (R version 4.3.3). Functional category annotations were derived from the Mtb H37Rv genome feature (GFF) file downloaded from Mycobrowser (version 5). Genes were assigned to Mycobrowser “Functional_Category” terms by parsing the corresponding attribute field in the GFF file. Genes were defined as DE using a false discovery rate (FDR) threshold of 0.05 and an absolute log_2_FC of >1. Genes with an FDR of <0.05 irrespective of fold change were analyzed in parallel.

For each functional category, enrichment was assessed by a two-sided Fisher’s exact test comparing the proportion of DE genes within the category to that in the genome-wide background. Categories with no annotated genes were excluded. For each term, the odds ratio, *P* value, and Benjamini–Hochberg-adjusted FDR were calculated. Enrichment direction (enriched or depleted) was assigned based on the relative proportion of DE genes in each category compared to the background.

Results were visualized using a custom ggplot2-based dot panel, in which categories were ranked by FDR and displayed with point size proportional to –log_10_(FDR) and color denoting enrichment direction. Output tables including enriched categories, adjusted statistics, and DE gene membership were exported as Excel workbooks using the openxlsx package. Heatmaps show *z*-scored log-normalized transcripts per million. Each column represents one host donor. Heatmaps are hierarchically clustered along both axes (by both donors and genes).

## Data Availability

RNA-seq data have been deposited in the GEO under accession number GSE330455. All data needed to evaluate the conclusions in the paper are present in the paper and/or the supplemental material.

## SUPPLEMENTAL MATERIAL

Figure S1 msphere.00141-26-s0001.tif *M. tuberculosis* forms ILI during mouse macrophage infection but not in human macrophages.

Figure S2 msphere.00141-26-s0002.tif *M. tuberculosis* can form ILIs at the different growth stages.

Figure S3 msphere.00141-26-s0003.tif Immortalized murine macrophage *irg1* and *nos2* gene knockout confirmation.

Figure S4 msphere.00141-26-s0004.tif Knockout of *M. tuberculosis* ESX-1 secretion system abolishes ILI formation during murine macrophage infection.

Figure S5 msphere.00141-26-s0005.tif Knockdown of DGAT1 or PLIN2 permits ILI formation in Mtb in human THP-1 macrophages.

Figure S6 msphere.00141-26-s0006.tif Human Cas9-expressing THP-1 macrophages DGAT1 and PLIN2 gene knockdown confirmation.

Supplemental material msphere.00141-26-s0007.docx Supplemental figure legends.

Table S1 msphere.00141-26-s0008.xlsx Mtb differential gene expression analysis in human monocyte-derived macrophages versus murine bone marrow-derived macrophages.

ASM does not own the copyrights to Supplemental Material that may be linked to, or accessed through, an article. The authors have granted ASM a non-exclusive, world-wide license to publish the Supplemental Material files. Please contact the corresponding author directly for reuse.
