# Supplementary figures and images for: Systems analysis reveals alternate metabolic states adopted by Mycobacterium tuberculosis across species
Source: mSphere. 2026 Jun 29;11(7):e00141-26. doi: 10.1128/msphere.00141-26 (PMC13410760; doi:10.1128/msphere.00141-26)

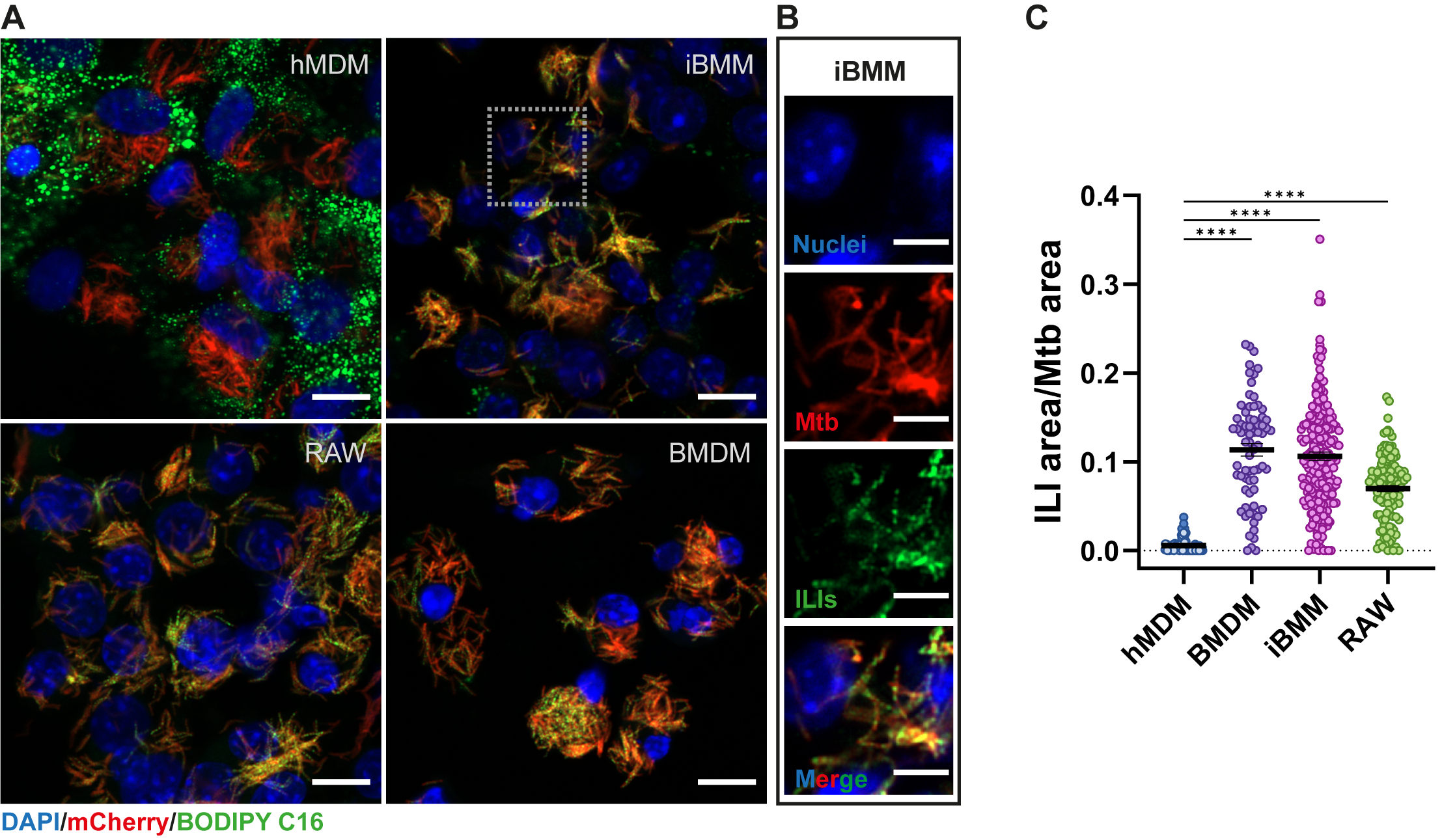

Supplement: Figure S1 — M. tuberculosis forms ILI during mouse macrophage infection but not in human macrophages. [file msphere.00141-26-s0001.tif]

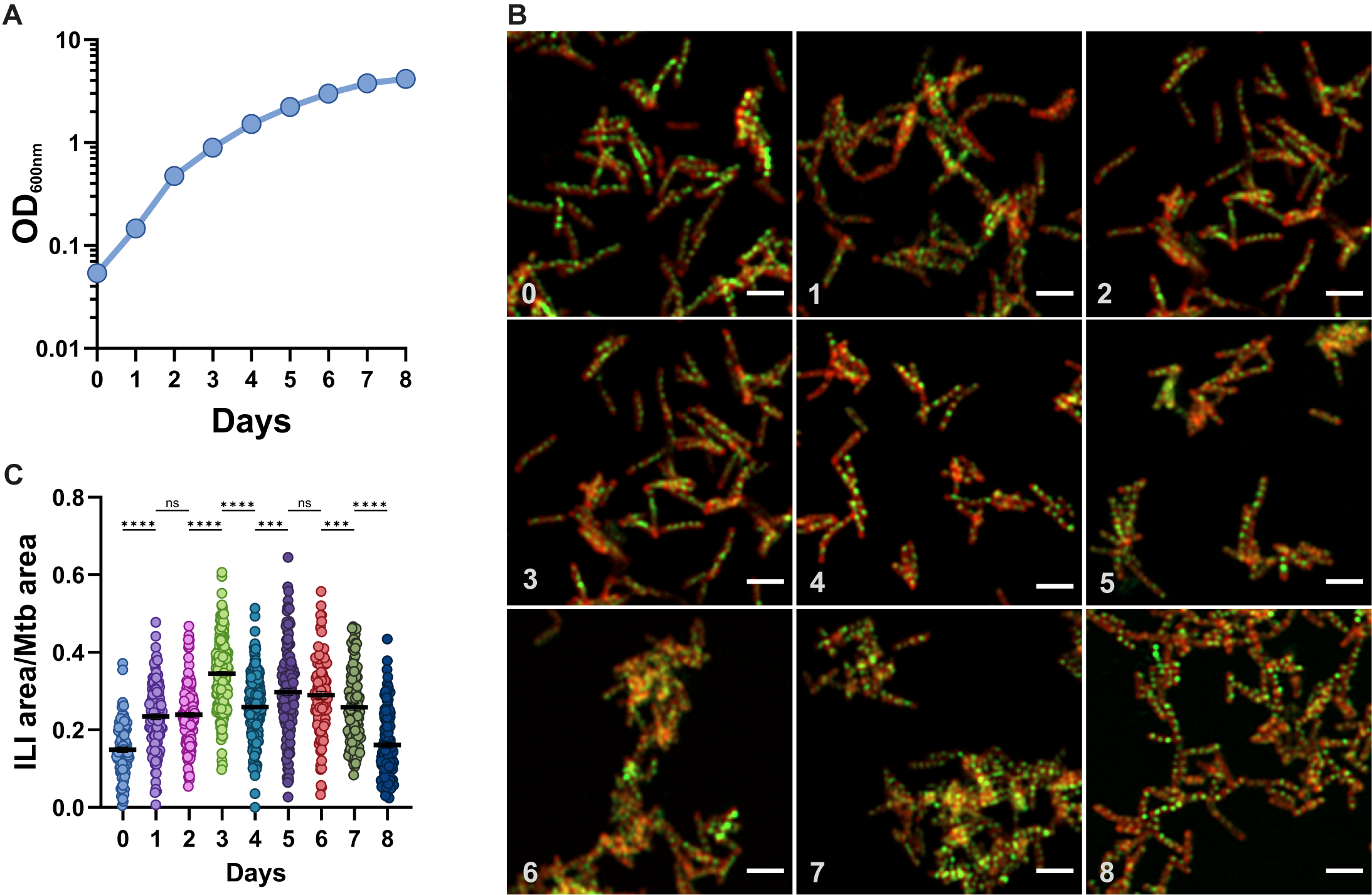

Supplement: Figure S2 — M. tuberculosis can form ILIs at the different growth stages. [file msphere.00141-26-s0002.tif]

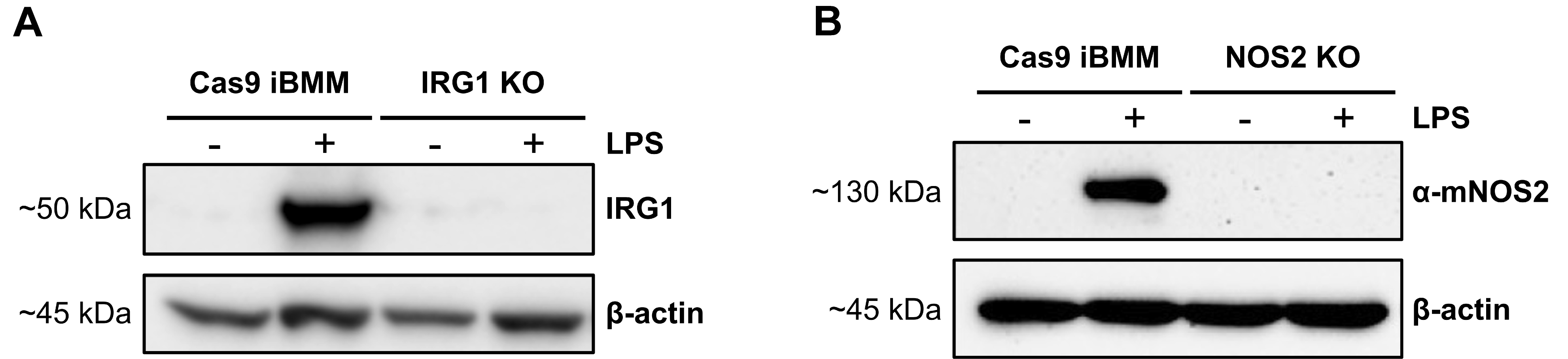

Supplement: Figure S3 — Immortalized murine macrophage irg1 and nos2 gene knockout confirmation. [file msphere.00141-26-s0003.tif]

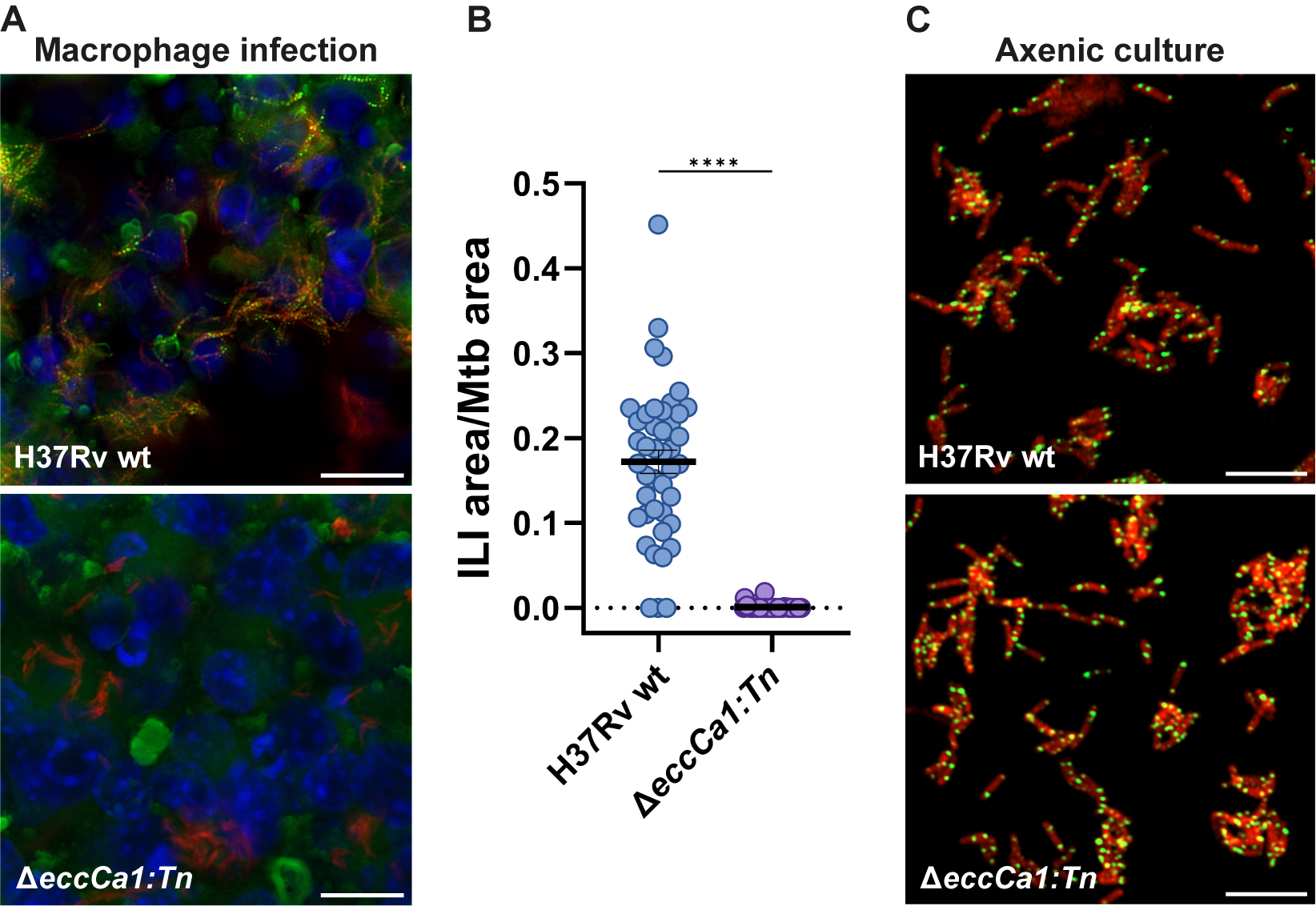

Supplement: Figure S4 — Knockout of M. tuberculosis ESX-1 secretion system abolishes ILI formation during murine macrophage infection. [file msphere.00141-26-s0004.tif]

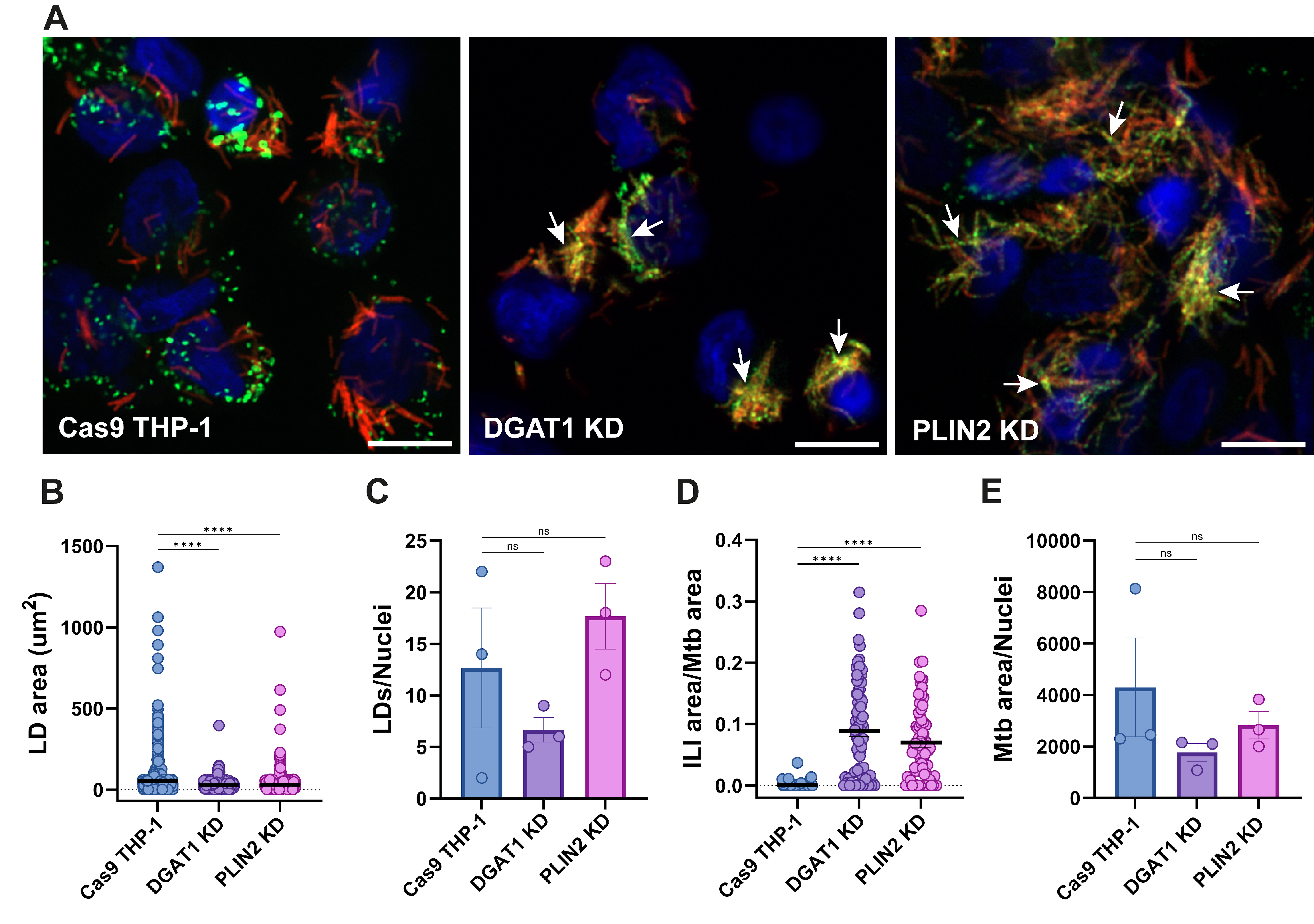

Supplement: Figure S5 — Knockdown of DGAT1 or PLIN2 permits ILI formation in Mtb in human THP-1 macrophages. [file msphere.00141-26-s0005.tif]

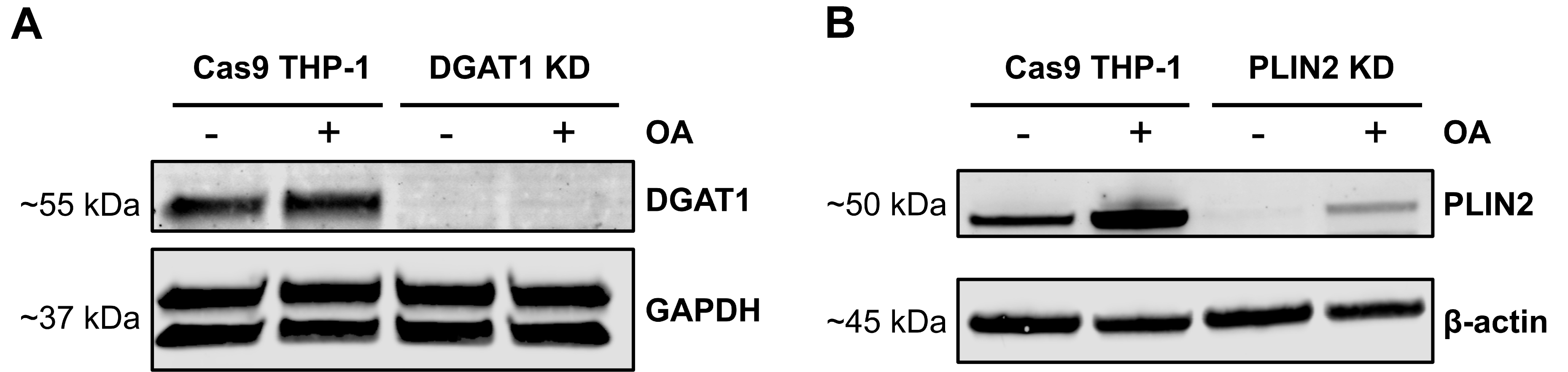

Supplement: Figure S6 — Human Cas9-expressing THP-1 macrophages DGAT1 and PLIN2 gene knockdown confirmation. [file msphere.00141-26-s0006.tif]
