# Supplementary material for: Systems analysis reveals alternate metabolic states adopted by Mycobacterium tuberculosis across species
Source: mSphere. 2026 Jun 29;11(7):e00141-26. doi: 10.1128/msphere.00141-26 (PMC13410760; doi:10.1128/msphere.00141-26)
Supplement: Supplemental material — Supplemental figure legends. [file msphere.00141-26-s0007.docx]

**SUPPLEMENTAL FIGURE LEGENDS**

**Supplemental Figure 1. *M. tuberculosis* forms intracellular lipid inclusions (ILI) during mouse macrophage infection but not in human macrophages.** **(A)** Human monocyte-derived macrophages (hMDMs) and three murine macrophage types—bone marrow-derived macrophages (BMDMs), immortalized BMDMs (iBMMs), and RAW264.7 macrophages (RAWs)—were infected with cytosolic mCherry-expressing Mtb Erdman for 24 h (MOI = 3 for hMDMs; MOI = 10 for murine macrophages). Following infection, neutral lipids were labeled with BODIPY C16 (8 h pulse, 1 h chase), samples were fixed with paraformaldehyde (PFA), treated with 60% isopropanol to reduce cytoplasmic background fluorescence, and stained with DAPI. Samples were visualized by Airyscan fluorescence microscopy. Scale bars, 10 µm. **(B)** Magnified insets from (A) (iBMMs, gray dashed box) showing nuclei (DAPI, blue), mCherry-labeled Mtb (red), and BODIPY C16-labeled ILIs (green) in separate channels and merged images. Scale bars, 5 µm. **(C)** Quantification of ILI fluorescence from (A), shown as ILI area / Mtb area ± SEM (N = 3) (**** p<0.0001; two-way ANOVA with Dunnett’s multiple comparisons test). hMDM data are color-coded to indicate independent donors.

**Supplemental Figure 2. *M. tuberculosis* can form ILIs at the different growth stages.** **(A)** Growth curve of mCherry-expressing Mtb Erdman cultured in 7H9^OADC^ medium, monitored by OD_600_. **(B)** At each day (0–8), cultures were assayed for ILI formation by labeling neutral lipids with BODIPY C16 (4 h pulse, 1 h chase) and visualized by Airyscan fluorescence microscopy. Scale bars, 2 µm. **(C)** Quantification of ILI signal from (B), shown as ILI area / Mtb area ± SEM (N = 3) (ns [not significant], *** p=0.0002, **** p<0.0001; two-way ANOVA with Dunnett’s multiple comparisons test).

**Supplemental Figure 3. Immortalized murine macrophage *irg1* and *nos2* gene knockout confirmation by Western blotting.** Total proteins were isolated from immortalized murine bone marrow-derived macrophages (iBMMs) expressing Cas9, or knockout (KO) mutants in Irg1 **(A)** or Nos2 **(B)**, plus/minus stimulation with 10 ng/mL of lipopolysaccharide (LPS). Western blotting was performed for total IRG1 (A) and ɑ-mNOS2 (B). Approximate molecular weights are annotated according to estimates for PageRuler Plus Prestained Protein Ladder, 4-12% Bis-Tris gel, MES running buffer. β-actin is shown as a protein loading control (bottom). Blots are representative of at least three independent experiments.

**Supplemental Figure 4. Knockout of *M. tuberculosis* ESX-1 secretion system abolishes ILI formation during murine macrophage infection.** **(A)** Immortalized murine macrophages (iBMMs) were infected with cytosolic mCherry-expressing Mtb H37Rv wild type (wt) or ESX-1 mutant (*eccCa1:Tn*) for 24 h (MOI = 10). After infection, 8 h BODIPY C16 pulse-1h chase experiments were performed. Cells were fixed, stained with DAPI, and visualized using Airyscan fluorescence microscopy. Scale bars, 10 µm. **(B)** Quantification of ILI signal from (A), presented as ILI area / Mtb area ± SEM (N = 3) (**** p<0.0001; unpaired t test with Welch’s correction). **(C)** mCherry-expressing Mtb H37Rv wt and *eccCa1:Tn* strains were cultured axenically in 7H9^OADC^ medium and labeled with BODIPY C16 (4 h pulse, 1 h chase) at OD₆₀₀ ≈ 1. Samples were fixed and visualized by Airyscan fluorescence microscopy. Scale bars, 5 µm. All data represent at least three independent experiments.

**Supplemental Figure 5. Knockdown of DGAT1 or PLIN2 permits ILI formation in Mtb in human THP-1 macrophages. (A)** Cas9-expressing human THP-1 macrophages or diacylglycerol acyltransferase 1 (DGAT1) or perilipin 2 (PLIN2) knockdown (KD) mutants were infected for 24h with cytosolic mCherry-expressing Mtb Erdman at MOI = 10. Following infection, neutral lipids were labelled with BODIPY C16 (8 h pulse, 1 h chase), cells were fixed, nuclei were stained with DAPI, and samples were imaged using Airyscan fluorescence microscopy. White arrows indicate Mtb containing ILIs. Scale bars, 10 µm. **(B–D)** Quantification of (B) lipid droplet (LD) area (µm²), (C) LD number per nucleus, and (D) ILI area per Mtb area from images in (A). Data are presented as mean ± SEM (N = 3) (C) or as ILI area / Mtb area ± SEM (N = 3) (D). **(E)** Mtb area per nucleus ± SEM (N = 3) (ns [not significant], **** p<0.0001; two-way ANOVA with Dunnett’s multiple comparisons test).

**Supplemental Figure 6. Human Cas9-expressing THP-1 macrophages DGAT1 and PLIN2 gene knockdown confirmation by Western-blot.** Total protein was isolated from human THP-1 macrophages expressing Cas9 (parental line), or DGAT1 **(A)** or PLIN2 **(B)** knockdown (KD) lines with or without treatment with 250 µM of oleic acid (OA). Western blotting was performed for total DGAT1 (A) or PLIN2 (B). Overexpression of PLIN2 under OA treatment can be observed in (B). Approximate molecular weights are annotated according to estimates for PageRuler Plus Prestained Protein Ladder, 4-12% Bis-Tris gel, MES running buffer. As protein loading controls, GAPDH is shown for DGAT1 in (A) (bottom) and β-actin is shown for PLIN2 in (B) (bottom). Samples shown in blots are representative of at least two replicates.
